# Study of machine learning techniques for outcome assessment of leptospirosis patients

**DOI:** 10.1038/s41598-024-62254-1

**Published:** 2024-06-17

**Authors:** Andreia Ferreira da Silva, Karla Figueiredo, Igor W. S. Falcão, Fernando A. R. Costa, Marcos César da Rocha Seruffo, Carla Cristina Guimarães de Moraes

**Affiliations:** 1 Laboratory of Zoonoses and Public Health - Federal University of Para, Av. dos Universitários - Jaderlândia, Belém, PA 68746-360 Brazil; 2 Department of Informatics and Computer Science, Institute of Mathematics and Statistics, Rio de Janeiro State University, Rua São Francisco Xavier, 524, Rio de Janeiro, 20550-013 Brazil; 3 Federal University of Para, R. Augusto Corrêa, 1 - Guamá, Belém, 66075-110 Brazil

**Keywords:** Leptospirosis, Data mining, Machine learning, Desfecho, Simulator, Decision tree, Predictive medicine, Signs and symptoms

## Abstract

Leptospirosis is a global disease that impacts people worldwide, particularly in humid and tropical regions, and is associated with significant socio-economic deficiencies. Its symptoms are often confused with other syndromes, which can compromise clinical diagnosis and the failure to carry out specific laboratory tests. In this respect, this paper presents a study of three algorithms (Decision Tree, Random Forest and Adaboost) for predicting the outcome (cure or death) of individuals with leptospirosis. Using the records contained in the government National System of Aggressions and Notification (SINAN, in portuguese) from 2007 to 2017, for the state of Pará, Brazil, where the temporal attributes of health care, symptoms (headache, vomiting, jaundice, calf pain) and clinical evolution (renal failure and respiratory changes) were used. In the performance evaluation of the selected models, it was observed that the Random Forest exhibited an accuracy of 90.81% for the training dataset, considering the attributes of experiment 8, and the Decision Tree presented an accuracy of 74.29 for the validation database. So, this result considers the best attributes pointed out by experiment 10: time first symptoms medical attention, time first symptoms ELISA sample collection, medical attention hospital admission time, headache, calf pain, vomiting, jaundice, renal insufficiency, and respiratory alterations. The contribution of this article is the confirmation that artificial intelligence, using the Decision Tree model algorithm, depicting the best choice as the final model to be used in future data for the prediction of human leptospirosis cases, helping in the diagnosis and course of the disease, aiming to avoid the evolution to death.

## Introduction

Leptospirosis is a bacterial disease that affects people all over the world, as well as domestic and wild animals^1,2^. When infected, these animals carry the pathogen in their renal tubules, releasing it through their urine, spreading the bacteria in the environment, allowing the infection of other beings when they come into contact with contaminated water and mud^1–4^.

This disease has been underestimated because it affects populations with socioeconomic vulnerability, living in areas without basic sanitation, especially during rainy seasons^1,3,4^. Moreover, its symptoms are similar to those of other diseases that occur during the same seasonal period^1^.

The highest occurrence of leptospirosis in Brazil has been recorded in the more developed regions (southeast and south), which are places with larger populations and greater access to health care^1,5–7^. In the northern region, although the climate and biological diversity are more conducive to the maintenance of the bacterium, the recording of cases of leptospirosis is lower, intensified by the difficulty in diagnosis and inefficient clinical care^5,8^. This limitation, besides having a direct impact on the number of confirmed cases in the region, makes it difficult for public health organizations to recognize cases and affects the development of policies aimed at eradicating the disease^9–11^.

The disease presents symptoms similar to other diseases, such as influenza, dengue, typhoid fever, rickettsiosis, aseptic meningitis, hepatitis, and malaria, which makes the correct diagnosis more difficult and agile, resulting in inadequate treatment and even death in more severe cases^1,12^. The transmission is associated with environmental factors such as the occurrence of floods that favor the contact of humans with the excreta of reservoirs^4^. The penetration of the microorganism in the host happens through the skin with lesions, or in the intact skin when immersed in water for a long time, or through the mucous membranes^3^.

In Brazil, public health planning is based on the records contained in government systems, such as the National System of Aggressions and Notification (SINAN, in Portuguese), where the notifications of cases (suspected, confirmed and unconfirmed) of diseases monitored by the Ministry of Health are recorded^13^.

Although these platforms also contribute to the advancement of health research, there are structural problems in their organization, such as missing values, data redundancy, and underreporting of cases, especially for leptospirosis^14^. These conditions reinforce the need to use Artificial Intelligence (AI) models to formulate solutions related to prediction, given the efficiency of learning models and the large number of characteristics considered in the health sector.

Predictive models from AI allow the identification of patterns, considering variables of various typologies, such as socioeconomic, demographic, social, clinical among others^15^. This approach is capable of profiling individuals who could possibly develop a favorable picture of some syndrome^16^. Moreover, with the aid of AI it is possible to apply learning models from a large volume of data, generating solutions to aid diagnosis, treatment and clinical follow-up, for example^17–21^.

Due to the above, this study aims to propose a model that acts in the identification of variables that most contributed to the outcome of cure or death of the patient with leptospirosis in the state of Para (2007-2017), from the implementation of data mining techniques for the development of model based on three classification algorithms (Decision Tree, Random Forest and Adaboost) and their machine learning performance metrics, in order to identify in advance which variables are more sensitive to the outcome of leptospirosis cases.

## Methods

### Data source

The data for this research is provided by the Department of Health of the State of Pará, Brazil, in accordance with Law No. 12.527, of November 18, 2011, in the form of aggregated information, without the possibility of individual identification. This secondary database can also be requested in person by the aforementioned sector or through the Citizen Information Service (SIC).

### Ethics Committee

The research was not submitted to the Ethics Committee, as according to Art. 26 of the Resolution of the National Health Council of Brazil n$^{\circ }.$ 674 of May 6, 2022, reinforced by Article 1 of the Resolution of the National Health Council of Brazil n$^{\circ }.$ 510 of April 7, 2016, is exempt from consideration by the Committee of ethics in the CEP/Conep System, research that uses publicly accessible information, uses public domain information and uses information or data already available in aggregate form, without the possibility of individual identification.

### Data

The data source was collected from SINAN, referring to the 1,319 cases of leptospirosis of patients in the state of Pará, which evolved to death or cure of the disease in the period between the years 2007 to 2017 (10 years), kindly provided by the Health Secretariat of the State of Para (SESPA, in Portuguese). Patients’ personal information was not used for confidentiality purposes.

The database used contained information on leptospirosis patients at different stages, with different times between the presentation of the first symptoms and medical care, even presenting initial symptoms similar to those of other illnesses that generally occur in the same period of the year (influenza, dengue fever, typhoid fever, rickettsioses, aseptic meningitis, hepatitis, and malaria).

**Figure 1:**
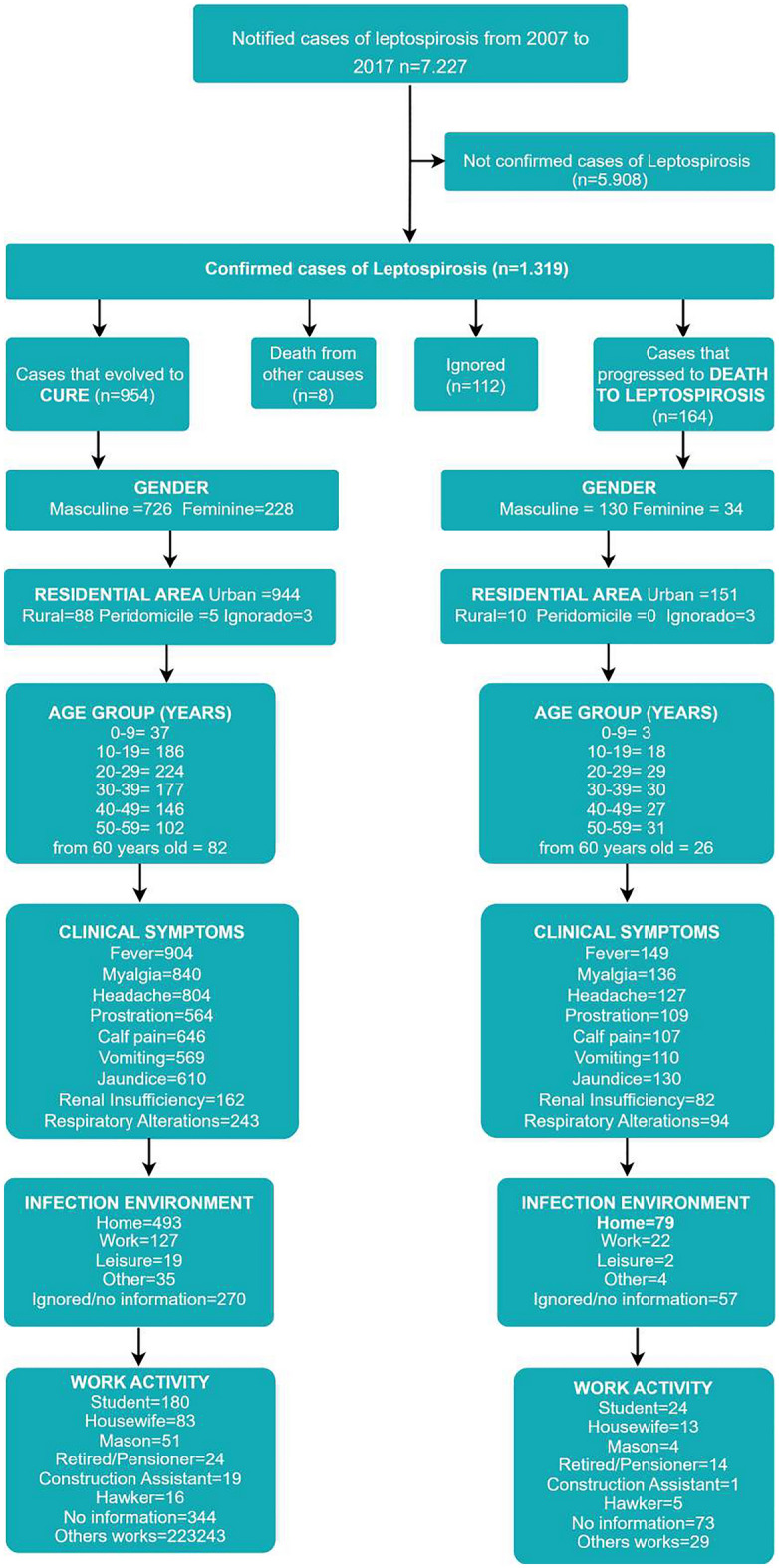
Inclusion and exclusion criteria flowchart.

In Figure 1 demonstrate that the only exclusion criterion was confirmation of the disease in patients with leptospirosis that evolved to cure or death. The same figure also present the frequency of some attributes of these groups:

### Database processing and preliminary attribute selection

The database obtained had a large number of attributes, and only those that dealt with specific characteristics of the disease were selected. During the process of extracting the data of interest, it was necessary to use a Data Science technique to manipulate all the information, the Exploratory Data Analysis (EDA), used to visualize the main characteristics, identifying insights and possible outputs of the data set. For the methodological process employed, the order of the steps for processing the input dataset was established. Initially, the SINAN database was analyzed, where the data of interest were chosen, which underwent pre-processing, and prediction by machine learning models, and later the results were evaluated according to the objective of the research, corroborating [23].

The choice of specific attributes was made to facilitate their application in predicting leptospirosis in regions lacking the necessary infrastructure for prompt laboratory diagnosis, a scenario often observed in numerous municipalities within the Amazon.

### Selection of the attributes of interest

Initially, 15 (fifteen) attributes, contained in Table 1, were selected for analysis of the patient’s outcome (death or cure). Before starting the data preprocessing process, it was essential to eliminate attributes that were not relevant to the object of the research, which was to investigate and evaluate models based on Machine Learning to infer the outcome of patients with leptospirosis.

**Table 1:** Attributes of interest.

Attribute	Number of missing values
Time elapsed between the appearance of the first symptoms and medical attention	22
Time elapsed between medical attention and hospital admission	270
Time elapsed between onset of first symptoms and first ELISA sample collection	216
Fever	30
Myalgia	38
Headache	49
Prostration	90
Calf Pain	81
Vomiting	63
Jaundice	59
Renal Insifficiency	110
Respiratory Alterations	85
Contact With Flood Water or Mud	175
Place With Signs of Rodents	182
Garbage / Debris	210

In this case, after evaluation, it was found that the attributes: contact with flood water or mud, place with signs of rodents, and garbage/debris could contribute to the diagnostic analysis, but not to the outcome evaluation, since it was already found that the patients were ill during the period studied.

After this evaluation, a new attribute selection was performed, keeping the following attributes from Table 1: elapsed time (in days) between first symptoms and medical care (T_first_symptoms_medical_attention), elapsed time (in days) between first symptoms and sample collection for the ELISA laboratory test (T_first_symptoms_sample_collection_ELISA), time elapsed (in days) between medical care and hospitalization (T_medical_attention_hospitalization), presentation of symptoms (headache, vomiting, jaundice, renal insufficiency, and respiratory changes).

### Preprocessing of attributes

For the remaining attributes it was observed the amount of missing values, a factor considered important for determining the attributes of interest. Missing values can be derived from several failures during the collection and storage of information, loss of data in the system or even human error of filling in the hosting platform. Thus, patient records that had more than 5 missing value attributes were eliminated, resulting in 1,120 records, accounting for only 164 death records in relation to the 956 cured patients.

Due to the unbalance of the base, in the pre-processing stage, it was mandatory to use methods to perform the balancing and the best procedure to adjust the large amount of missing values in several attributes of the base (Table 1), such as time elapsed between medical care and hospital admission (270), time elapsed between the onset of the first symptoms and the collection of the first sample for ELISA (216), fever (30), myalgia (38), headache (49), prostration (90), calf pain (81), vomiting (63), jaundice (59), renal insufficiency (110), respiratory changes (85).

Considering the small and unbalanced database, keeping records with missing and undetermined values (treated as undetermined) was necessary, and augmentation using SMOTE technique to balance the cure or death bases. Thus, the feature_INDETERMINED (binary) attribute was created to indicate when the value of a given attribute for a record was unknown (in this case, the feature_INDETERMINED attribute would be set to 1). If the attribute was known (yes = 1 or no = 0), the feature_INDETERMINED attribute of that record will be 0, indicating that the attribute information is known.

**Table 2:** Attributes and their values analyzed in the work.

Attribute	Values observed in the base
Time_first_symptoms_medical_attention	Numeric
Time_first_symptoms_ELISA_sample_collection	Numeric
Medical_attention_hospital_admission_time	Numeric
Fever	{0,1}
Myalgia_INDETERMINATE	{0,1}
Myalgia_TRUE	{0,1}
Headache_INDETERMINATE	{0,1}
Headache_TRUE	{0,1}
Prostration_INDETERMINATE	{0,1}
Prostration_TRUE	{0,1}
Calf_pain_INDETERMINATE	{0,1}
Calf_pain_TRUE	{0,1}
Vomiting_INDETERMINATE	{0,1}
Vomiting_TRUE	{0,1}
Jaundice_INDETERMINATE	{0,1}
Jaundice_TRUE	{0,1}
Renal insufficiency_INDETERMINATE	{0,1}
Renal insufficiency_TRUE	{0,1}
Respiratory_alterations_INDETERMINATE	{0,1}
Respiratory_alterations_TRUE	{0,1}

Due to the problems related to the occurrence of unbalance between the classes death and cure, balancing was performed using the SMOTE method^22^. To apply this method, attributes were added to discriminate the cases of indeterminate information in the database. This is necessary for the categorical attributes that had missing values. Thus, at the end of this step, Table 2 shows the list of attributes considered and the values associated with each attribute. Note that only the attribute fever does not have an undetermined option.

The adjustment of the predictive model was performed by dividing the data set in holdout mode into training and test. The balanced sample was divided in a stratified way (with the same number of records for each class in the training and test files), and the test database was formed with records not artificially generated in the balancing process, and, for this reason, it was not possible to build a test database with more than 70 records (35 of each class, cure and death). For the training database, there were 890 records of cured patients and 890 deaths.

Next, CFS^23^ and ReliefF^24^ variable selection methods were used to indicate which variables might be less favorable to the outcome performance. The results of the descending order in terms of importance by the methods can be seen in Table 3.

**Table 3:** Ranking of importance of the variables according to ReliefF and CFS Selection methods.

CFS	ReliefF
Time_first_symptoms_medical_attention	Time_first_symptoms_ELISA_sample_collection
Time_first_symptoms_ELISA_sample_collection	Medical_attention_hospital_admission_time
Medical_attention_hospital_admission_time	Jaundice_TRUE
Headache_TRUE	Respiratory_alterations_TRUE
Vomiting_TRUE	Calf_pain_TRUE
Jaundice_INDETERMINATE	Vomiting_TRUE
Renal_insufficiency_TRUE	Renal_insufficiency_TRUE
Respiratory_alterations_TRUE	Headache_TRUE
	Prostration_TRUE
	Myalgia_TRUE
	Fever_TRUE
	Respiratory_alterations_INDETERMINATE
	Renal_insufficiency_INDETERMINATE
	Prostration_INDETERMINATE
	Calf_pain_INDETERMINATE
	Time_first_symptoms_medical_attention
	Jaundice_INDETERMINATE
	Vomiting_INDETERMINATE
	Headache_INDETERMINATE
	Myalgia_INDETERMINATE

To investigate the most suitable set of attributes, several configurations used in the classification models were evaluated, removing variables one by one, as can be seen in each row of Table 4. Except for the first experiment, which considered all attributes, i.e., did not remove any, all other experiments were created by suppressing attributes considered less important by the attribute selection methods that presented negative importances value in the ReliefF selection method ranking.

**Table 4:** Indication of the combinations of attributes that were removed from the database.

Experiment	Disregarding the attributes
1	–
2	Vomiting_INDETERMINATE, Headache_INDETERMINATE and Myalgia_INDETERMINATE
3	Jaundice_INDETERMINATE, Vomiting_INDETERMINATE, Headache_INDETERMINATE Myalgia_INDETERMINATE
4	Jaundice_INDETERMINATE, Vomiting_INDETERMINATE, Headache_INDETERMINATE, Myalgia_INDETERMINATE, Calf_pain_INDETERMINATE
5	Jaundice_INDETERMINATE, Vomiting_INDETERMINATE, Headache_INDETERMINATE, Myalgia_INDETERMINATE, Calf_pain_INDETERMINATE, Prostration_INDETERMINATE
6	Jaundice_INDETERMINATE, Vomiting_INDETERMINATE, Headache_INDETERMINATE, Myalgia_INDETERMINATE, Calf_pain_INDETERMINATE, Prostration_INDETERMINATE, Renal_insufficiency_INDETERMINATE
7	Jaundice_INDETERMINATE, Vomiting_INDETERMINATE, Headache_INDETERMINATE, Myalgia_INDETERMINATE, Calf_pain_INDETERMINATE, Prostration_INDETERMINATE, Renal_insufficiency_INDETERMINATE, Respiratory_alterations_INDETERMINATE
8	Jaundice_INDETERMINATE, Vomiting_INDETERMINATE, Headache_INDETERMINATE, Myalgia_INDETERMINATE, Calf_pain_INDETERMINATE, Prostration_INDETERMINATE, Renal_insufficiency_INDETERMINATE, Respiratory_alterations_INDETERMINATE Fever_TRUE
9	Jaundice_INDETERMINATE, Vomiting_INDETERMINATE, Headache_INDETERMINATE, Myalgia_INDETERMINATE, Calf_pain_INDETERMINATE, Prostration_INDETERMINATE, Renal_insufficiency_INDETERMINATE, Respiratory_alterations_INDETERMINATE, Fever_TRUE,Myalgia_TRUE
10	Jaundice_INDETERMINATE, Vomiting_INDETERMINATE, Headache_INDETERMINATE, Myalgia_INDETERMINATE, Calf_pain_INDETERMINATE, Prostration_INDETERMINATE, Renal_insufficiency_INDETERMINATE, Respiratory_alterations_INDETERMINATE, Fever_TRUE, Myalgia_TRUE, Prostration_TRUE
11	Jaundice_INDETERMINATE, Vomiting_INDETERMINATE, Headache_INDETERMINATE, Myalgia_INDETERMINATE, Calf_pain_INDETERMINATE, Prostration_INDETERMINATE, Renal_insufficiency_INDETERMINATE, Respiratory_alterations_INDETERMINATE, Fever_TRUE, Myalgia_TRUE, Prostration_TRUE, Headache_TRUE
12	Jaundice_INDETERMINATE, Vomiting_INDETERMINATE, Headache_INDETERMINATE, Myalgia_INDETERMINATE, Calf_pain_INDETERMINATE, Prostration_INDETERMINATE, Renal_insufficiency_INDETERMINATE, Respiratory_alterations_INDETERMINATE, Fever_TRUE, Myalgia_TRUE, Prostration_TRUE, Headache_TRUE, Renal_insufficiency_TRUE
13	Jaundice_INDETERMINATE, Vomiting_INDETERMINATE, Headache_INDETERMINATE, Myalgia_INDETERMINATE, Calf_pain_INDETERMINATE, Prostration_INDETERMINATE, Renal_insufficiency_INDETERMINATE, Respiratory_alterations_INDETERMINATE, Fever_TRUE, Myalgia_TRUE, Prostration_TRUE, Headache_TRUE, Renal_insufficiency_TRUE, Vomiting_TRUE

## Results

The study focused on the initial understanding of data related to the outcomes of leptospirosis cases through Exploratory Data Analysis. The objective of this analysis was to identify patterns, trends, and outliers before applying specific learning techniques. This approach provided an in-depth insight into the dataset, laying a solid foundation for the implementation of subsequent techniques.

**Figure 2:**
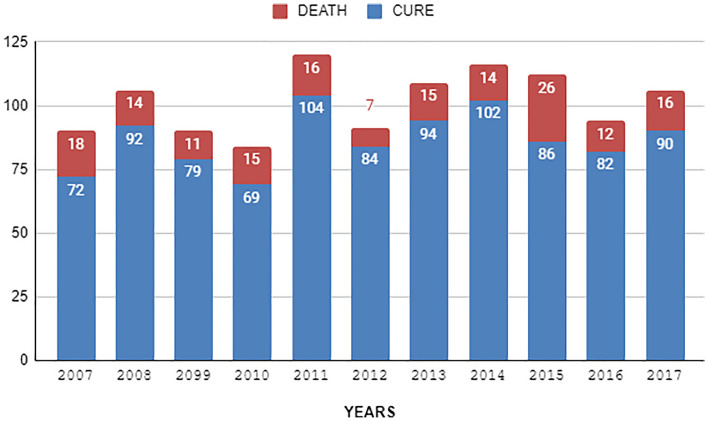
Quantitative of confirmed cases that evolved into cure or death.

The implementation of exploratory analysis is a procedure that precedes the application of learning models, as illustrated in Figure 2, detailing the entire process. Initially, the mortality rate was 14.66% concerning the total cases, making it imperative to employ the SMOTE Model to balance the dataset. This process is crucial in the context of machine learning to address imbalances in data classes, enhancing model performance, and mitigating overfitting. In addition, the use of augmentation, through the SMOTE technique, on the training data avoids data replication, focusing on preserving the diversity and representativeness of the original data, allowing for realistic variations, improving the robustness and generalizability of the model, acting as an even better protection against overfitting.

Mortality rates among individuals with leptospirosis from 2007 to 2017 reveal interesting trends, as shown in Figure 2. In 2007, the recovery rate was at 80%, with a mortality rate of 20%. Over the years, cure rates showed a steady increase, reaching a peak in 2011, reaching 87%, while mortality rates decreased. The following years exhibited variations in recovery and mortality rates, illustrating the evolution of patterns over the analyzed period.

### Definition of machine learning (ML) models

The ML models chosen to evaluate the outcomes were Random Forest, Adaboost and Decision Tree. The motivations for choosing these models were: the use of these models in similar studies and the good results obtained, when compared to various models that were also tested. These models have been widely used for this purpose, with the implementation of Python 3.7.6 and the libraries scikit-learn, numpy, pandas and matplotlib.

### Model analysis

The results obtained were compared based on performance evaluation metrics, which were: precision, recall, f1-score, MCC (Matthews correlation coefficient) and ROC (Receiver Operating Characteristics), besides the confusion matrix that is an indicator of identification of all four types of classification performance (True Positive - TP, False Negative - FN, False Positive - FP and True Negative - TN) of the binary classification model. Note that one of the main goals of the classifiers is to maximize the instances of TP (patients who will die as indicated by the algorithm and who actually died) and TN (patients who will be cured as indicated by the algorithm and who actually are cured), which represent the test confusion matrix of the models used. For the FP and FN of the confusion matrix, we aimed to minimize the values obtained, since they represent errors in the classification of possible leptospirosis outcomes.

After investigating methods applied to the data in the preprocessing phase, the results obtained in the experiments showed that some supervised machine learning models produce a good classification depending on the attributes and hyperparameters used. The choice of hyperparameters is also a task that directly impacts the performance of the classification model, so its definition was supported by the Grid Search method (it exhaustively combines the values listed for each algorithm, evaluating all the models resulting from these combinations). Table 5 presents the hyperparameters investigated for each algorithm, which allowed adjusting the training of the models used.

**Table 5:** Hyperparameters evaluated in the algorithms.

Algorithm	Hyperparameters
Decision tree	depth= {“max_depth”:[3,4, 5, 6, 7, 8, 9, 10, 11, 12, 13, 14, 15], “min_samples_split”:[2, 4, 8, 12, 16], “min_samples_leaf”: [2, 3, 4, 5, 6, 7, 8], “criterion”: [ ‘gini’, ‘entropy’] }
Random forest	depth= {“max_depth”:[3,4, 5, 6, 7, 8, 9, 10], “min_samples_split”:[2, 4, 8, 12, 16], ‘n_estimators’: [50, 100, 150], ‘criterion’: [‘gini’, ‘entropy’], “max_features’: [‘auto’, 2, 3, 4, 6, 8, 10 ,11], ‘min_samples_leaf’ : [2, 3, 4, 5, 6, 7, 8]}
Adaboost	depth= {“max_depth”:[1,2,3], learning_rate = [0.05, 0.1, 0.2, 0.3, 0.4, 0.5, 0.6, 0.7, 0.8, 0.9, 1] ‘n_estimators’: [50, 100, 150, 200], ‘min_samples_leaf’ : [1, 2, 3, 4, 5, 6, 7, 8, 9, 10]}

For the Decision Tree, values for each hyperparameter of the algorithm were evaluated: maximum depth of the tree—integer values in the interval {3,15}, the minimum number of samples for a node—integer values in the interval {2, 15}, the minimum number of leaves—integer values in the interval {2,8} and the impurity metrics “gini” and “entropy”. Thus, to identify the best configuration of these hyperparameters, an exhaustive search is performed combining all possible values of these attributes, of which 840 different models for Decision Tree, 19200 for Random Forest, and 1200 for Adboost.

For Random Forest the following hyperparameter possibilities were specified to be investigated: maximum depth {3,10} (integer values in the interval), the minimum number of samples for a node with integers in the interval {2, 16}, the number of trees used were considered the values of the dataset {50, 100 and 150}, attribute evaluation criteria “gini” and “entropy”, and the maximum number of features to be randomly drawn for the evaluation criteria of each attribute to be measured, ranging from values in the interval {2, 11}.

As for Adaboost, the following values were checked for hyperparameters maximum depth:1,2,3, learning rate {0.05, 0.10, 0.20, 0.30, 0.40, 0.50, 0.60, 0.70, 0.80, 0.90, 1.00}, number of “weak” estimators: {50, 100, 150, 200} and minimum number of samples for a leaf: {1, 2, 3, 4, 5, 6, 7, 8, 9, 10}.

The ML models were evaluated based on accuracy. The results shown in Table 6 (training base) and Table 7 (validation base), demonstrate the values obtained with the accuracy in the respective models. In both tables, the first column indicates the evaluated algorithms and the following 13 columns indicate each of the evaluated input attribute configurations (Table 4), considering the importance of the variables according to the ReliefF and CFS Selection methods (Table 3).

In the performance of the training set (Table 6), Random Forest showed the best accuracy result (90.81) and superior results compared to the other models evaluated in the training set. Experiment 8 is about the removing the set of attributes: Jaundice_INDETERMINATE, Vomiting_INDETERMINATE, Headache _INDETERMINATE, Myalgia_INDETERMINATE, Calf_pain_INDETERMINATE, Prostration_INDETERMINATE, Renal_insufficiency_INDETERMINATE, Respiratory_alterations_INDETERMINATE and Fever_TRUE.

Then, the best composition of attributes to be considered was also identified, highlighting in Experiment 8: time first symptoms medical attention, time first symptoms ELISA sample collection, medical attention hospital admission time, myalgia TRUE, headache TRUE, prostration TRUE, calf pain TRUE, vomiting TRUE, jaundice TRUE, renal insufficiency TRUE and respiratory alterations TRUE.

**Table 6:** Performance evaluation results.

Algorithm	1	2	3	4	5	6	7	8	9	10	11	12	13
Random forest	90.76	90.64	90.76	90.53	90.76	90.59	90.70	90.81	90.76	89.36	89.47	88.23	87.62
Adaboost	80.84	80.84	80.84	80.84	80.84	80.84	80.84	80.84	80.84	80.84	80.84	79.27	78.66
Decision tree	86.27	86.27	86.27	86.39	86.33	86.33	85.71	86.44	86.61	62.86	85.32	84.20	83.03

Experiment with attributes removed from the training set. Performance evaluation results, considering the Training set with the ML—Correctly Classified Instances—Accuracy algorithms. Combination of Attributes in the Training Set Algorithm 1 2 3 4 5 6 7 8 9 10 11 12 13 Random Forest, Adaboost and Decision Tree. Random Forest showed the best accuracy result (90.81).

As the model should be chosen considering the metrics obtained in the validation set, the results obtained with the validation basis, shown in Table 7, show that Decision Tree obtained the best result for accuracy (74.29) among the combinations of attributes evaluated (Table 4). Experiment 10 is about removing the set of attributes: Jaundice_INDETERMINATE, Vomiting_INDETERMINATE, Headache_INDETERMINATE, Myalgia_INDETERMINATE, Calf_pain_INDETERMINATE, Prostration_INDETERMINATE, Renal_insufficiency_INDETERMINATE, Respiratory_alterations_INDETERMINATE, Fever_TRUE, Myalgia_TRUE, Prostration_TRUE.

Thus, in addition to searching for the best hyperparameters per algorithm and the best algorithm among those evaluated, the best composition of attributes to be considered was also identified, highlighting in Experiment 10 that the attributes: time first symptoms medical attention, time first symptoms ELISA sample collection, medical attention hospital admission time, headache, calf pain, vomiting, jaundice, renal insufficiency, respiratory alterations were considered to be the ones that most help discriminate leptospirosis defects in the context of the data provided by the SINAN system.

It should be noted that the search for the best attributes was based on the use of filter-type variable selection techniques (CFS and ReliefF) mentioned in the Selection of the attributes of interest subsection, which is below the “Methods” section.

Confusion matrices were also generated for each model obtained. The confusion matrix for the Decision Tree model (Table 8) showed the amount of True Positive - TP (patients who die as indicated by the algorithm and who actually died = 19), False Negative - FN (death x cure = 16), False Positive - FP (cure x death = 10) and True Negative - TN (patients who will be cured as indicated by the algorithm and who are actually cured = 25), indicating greater efficiency of the model for cases in which the outcome is cure (n = 16). The confusion matrix is an important metric for evaluating the performance of models generated by machine learning, because it is from it that the other metrics (precision, accuracy, revocation, among others) are generated.

**Table 7:** Performance evaluation results.

Algorithm	1	2	3	4	5	6	7	8	9	10	11	12	13
Random forest	61.43	62.86	64.29	61.43	62.86	62.86	64.23	60.00	58.57	60,00	62.86	58.57	54.23
Adaboost	71.43	71.43	71.43	71.43	71.43	71.43	71.43	71.43	71.43	71.43	71.43	71.43	70,00
Decision tree	60.00	58.57	58.57	58.57	58.57	58.57	58.57	58.57	57.14	74.29	62.86	54.29	58.57

Experiment with attributes removed from the training set. Performance evaluation results considering the Validation set of ML algorithms—Correctly Classified Instances—Accuracy. Combination of Attributes in the Training Set Algorithm 1 2 3 4 5 6 7 8 9 10 11 12 13 Random forest, Adaboost and Decision tree. We observe that Decision Tree obtained the best result for accuracy (74.29) in experiment 10.

As an ensemble learning technique that combines multiple decision trees to make more robust and accurate decisions, it can help reduce the overfitting that can occur in a single decision tree, often leading to better performance. Random Forest, with its voting mechanism, can provide a more robust assessment of the importance of features by building multiple independent trees, while AdaBoost can adjust the weight of observations to focus more on those that have been incorrectly classified.

Decision Trees can be more susceptible to overfitting, but depending on the hyperparameters configured, a Decision Tree can perform excellently on the test base, avoiding overfitting. In addition, because they are less sensitive to hyperparameters, Decision Trees, compared to Random Forest and AdaBoost, can be less susceptible to specific hyperparameters, making configuration simple and better performance on the test bed. Thus, if the most important features for the task are well represented in the first nodes of the Decision Tree model, the intrinsic ability of a Decision Tree to evaluate features may be enough to obtain good performance. A single Decision Tree can also have advantages for smaller data sets, as it is less susceptible to overfitting in small sets.

It is worth emphasizing that using a validation set aims to choose the best algorithm/model, choosing the one that has the lowest bias error and variance on a new set of data.

**Table 8:** Confusion Matrix Validation with the result of the Decision Tree model.

Validation confusion matrix	Expected	
Real	Death	Cure
Death	19	16
Cure	10	25

## Discussion

There are several works where the artificial intelligence techniques have been applied in the health area, presenting good results in the prediction and diagnosis of diseases caused by several etiological agents or organism dysfunctions. Among them: the classifier based on Convolutional Neural Network, using Google open source tools and TensorFlow for lesion recognition in leprosy^25^, obtaining 91.6% accuracy for detecting leprosy lesions of patients; diagnosis of heart diseases based on ML models, including Neural Networks, Support Vector Machine, Regression and Decision Tree for identifying disease-specific characteristics^26^; and classification algorithms (Random, Forest, Decision Trees, Extra Trees, Boosting of the stochastic gradient and Adaboost) to discover the features associated with the spread of dengue in the environment, as well as measuring fever, bleeding, myalgia, glandular flu, fatigue and the outcomes (positive/negative) of patients under evaluation^27^.

However, studies that use ML techniques applied to the diagnosis of human leptospirosis and the prediction of cases with outcome of death or cure of patients are still incipient. Among them, we have the case of^28^ that used clinically and epidemiologically confirmed cases of leptospirosis to predict cases using data mining classification algorithms. The algorithm used was JRIP, which obtained 84% sensitivity and 99% specificity using the data of only confirmed leptospirosis cases and a specificity of 99% when the database of confirmed dengue cases was used. Thus, the tested artificial intelligence techniques predicted the cases of leptospirosis, differentiating them from dengue, which is a febrile disease with similar symptoms, resulting in a tool to assist health professionals in leptospirosis endemic areas, in order to accelerate the diagnosis and treatment, minimizing deaths.

The researchers^29^, concerned with the similarity of symptoms and difficulty of laboratory diagnosis of infections, conducted a study to develop a decision-making tool to differentiate infections of dengue, malaria, leptospirosis and typhoid in a Malaysian hospital. A questionnaire was used as data for the physicians who assessed the need for the development of the decision-making tool, and 800 cases were collected regarding dengue, malaria, leptospirosis, and typhoid.

The data set was analyzed with the multinomial regression technique, machine learning model (multiclassification and binary classification). The multinomial logistic regression showed 60.7%, 62.5% and 66% predictability for dengue, malaria and leptospirosis, respectively. Similarly, the multiclassification machine learning model showed 55% to 60% predictability. Thus, the authors recommend machine learning techniques as a tool for disease prediction, with the goal of early detection of cases, and consequently better patient care.

So did the researchers^30^ who observed the need to insert artificial intelligence techniques to increase the efficiency and accuracy of Microscopic Agglutination Test (MAT) laboratory analyses. To do this, they built an algorithm for automatic identification of MAT, using machine learning to determine the positive cases in the microscopic agglutination images of the test, in addition to the use of support vector machine (SVM). The use of the techniques demonstrated the possibility of automating the analysis of MAT test images, as it obtained a sensitivity of 0.99 and specificity of 0.99 in confirming positive images.

It is noteworthy that artificial intelligence techniques have been frequently updated, providing greater reliability and efficiency in their application. The efficiency in the prediction and diagnosis of leptospirosis is an extremely important factor for health professionals and especially for the definition of effective treatment of the disease, reducing the possibility of evolution to death, as well as reducing the costs of health treatment by the public system.

## Conclusion

After evaluating the performance of the selected models according to the metrics obtained from the test set (not used to build the solution), it became clear that the Decision Tree represents the best choice as the final model to be used on future data.

Therefore, the use of artificial intelligence to predict the outcome of human leptospirosis cases can help health professionals in the diagnosis and course of the disease, avoiding costs and especially deaths. In addition, this study suggests improvements in the filling of leptospirosis databases in order to improve the prediction of the outcome of cases.

Thus, the methodology presented here allows reproducibility for other experiments and contributes to the state of the art, as it can guide researchers who undertake similar studies. Furthermore, it allows for an element of comparison, through the AI techniques presented here, as well as data science tools and methodology for standardization and knowledge extraction.

Although the data was specific to the occurrence of this disease in Brazil, a country with socio-economic deficiencies that favor the occurrence of this disease in an endemic way, the contributions of the work, either with the methodology or even with the best model identified, can be explored and used by other countries where Leptospirosis occurs. The group intends to expand its network of partners, looking for information and databases on this disease in countries that also suffer from Leptospirosis.

## Data Availability

The datasets utilized and/or analyzed in the current study can be found in the OSF Data repository and can be accessed via the link (https://osf.io/vse4t/?view_only=54e9252d993d474e89d575b612a6daaa).
